# Environmental Isolates of Multi-Azole-Resistant *Aspergillus* spp. in Southern Italy

**DOI:** 10.3390/jof4040131

**Published:** 2018-12-06

**Authors:** Laura Trovato, Guido Scalia, Maria Domina, Salvatore Oliveri

**Affiliations:** 1 U.O.C. Laboratory Analysis Unit, A.O.U. “Policlinico-Vittorio Emanuele”, 95123 Catania, Italy; lido@unict.it (G.S.); oliveri@unict.it (S.O.); 2 Department of Biomedical and Biotechnological Sciences, University of Catania, 95123 Catania, Italy; m.domina@tiscali.it

**Keywords:** azole resistance, *Aspergillus*, environment

## Abstract

Azole resistance in *Aspergillus* spp. has been increasingly reported worldwide. Acquired azole resistance is probably linked to environmental exposure to fungicides used in agriculture. We collected a total of 84 soil and leaf samples from eight farms in Southern Italy. *Aspergillus* isolates were tested for resistance to itraconazole, posaconazole, and voriconazole by the EUCAST method. Five out of 84 samples yielded *A. fumigatus* isolates: four of them were itraconazole-resistant and were identified as *A. fumigatus sensu stricto,* three of them were posaconazole-resistant, and two were also voriconazole-resistant. All three isolates harbored the TR_34_/L98H resistance mechanism, which was detected by DNA sequencing of the *cyp51A* gene. Fifteen out of 84 samples yielded *Aspergillus* spp. isolates and included 11 itraconazole-resistant isolates: *Aspergillus* section *Nigri* (9) and *Aspergillus* section *Flavi* (2). Our study reports for the first time the isolation of azole-resistant *A. fumigatus* harboring TR_34_/L98H mutation from the environment of Southern Italy. The present work provides a better understanding of the magnitude of the environmental spread of azole resistance in the context of a necessary effective surveillance program to improve the management of *Aspergillus-*related disease.

## 1. Introduction

*Aspergillus* spp. are ubiquitous saprophytic fungi that commonly occur in soil, water, and decaying vegetation, producing conidia which can be easily dispersed to the ambient air. Inhalation of such conidia, in association with certain risk conditions, can trigger aspergillosis, a group of several pulmonary diseases in humans, ranging from allergic bronchopulmonary aspergillosis (ABPA), chronic pulmonary aspergillosis (CPA), and aspergilloma to the most severe form, invasive aspergillosis (IA) [1,2]. Risk groups for aspergillosis mainly include immunocompromised subjects, such as patients with hematological malignancies, prolonged neutropenia or neutrophil disorders, pulmonary diseases, or solid-organ or hematopoietic stem cell transplantation [3,4,5]; those receiving prolonged high-dose corticosteroid therapy; and critically ill patients in intensive care units [6,7].

Triazole antifungal drugs (i.e., itraconazole, voriconazole, posaconazole, and isavuconazole) are the major compounds currently involved in the treatment and prophylaxis of aspergillosis [8]: itraconazole, the first orally active drug for aspergillosis, is commonly used in the treatment of chronic pulmonary aspergillosis and allergic conditions [9]; voriconazole, with its oral and intravenous availability, is the first-choice therapy for invasive aspergillosis [10]; posaconazole is preferred for the prevention of invasive aspergillosis in leukemia and bone marrow transplant patients [11]; and isavuconazole has only recently been approved for the treatment of invasive aspergillosis [12].

In recent years, the global emergence of azole-resistant isolates of *Aspergillus* spp. has been increasingly reported in patients under long-term antifungal treatment. However, azole-resistant isolates are also now being reported in azole-naïve patients with no known prior exposure to azole drugs, as well as in isolates from the environment [13]. The main resistance mechanism of the environmental isolates consists of a 34-base pair tandem repeat in the promoter region of the *cyp51A* gene, encoding the enzyme target of antifungal azoles and responsible for converting lanosterol to ergosterol via demethylation, in combination with a point mutation of the same gene leading to a substitution of leucine for histidine at codon 98 (TR_34_/L98H) [14]. Strong evidence supports the environmental origin of resistant *Aspergillus fumigatus* to medical triazoles following exposure to triazole fungicides used in agriculture (the so-called DMI, or demethylation inhibitor fungicides) [15,16,17,18]. In particular, the isolation of azole-resistant isolates of *Aspergillus* in azole-naïve patients with invasive aspergillosis as well as breakthrough aspergillosis in patients on azole prophylaxis would support the hypothesis of the acquisition of strains with dominant resistance mechanisms from the environment. Few data are available on *A. fumigatus* azole resistance in Italy [19,20], while azole resistance in non-*fumigatus Aspergillus* species has never been investigated. With the present study we aim to expand knowledge about the spread of environmental of TR_34_/L98H mutation in Italy with data from southern regions. We also report data on the prevalence of phenotypic azole resistance in non-*fumigatus Aspergillus* spp. isolated from the environment in Southern Italy.

## 2. Materials and Methods

### 2.1. Selection of Agricultural Areas and Enviromental Sampling

After a careful search of the agricultural zones with the largest production of greenhouse and open-field vegetables and use of triazole fungicides, we selected five farms in Sicily (two farms producing vegetables, two producing citrus fruits and cereals, and one producing cereals), two farms producing vegetables and cereals in Calabria, and one farm producing olive trees and cereals in Basilicata. These companies were located at a distance of 5–10 km from urban centers. Bromuconazole, cyproconazole, paclobutrazol, difenoconazole, hexaconazole, fenbuconazole, tetraconazole, tebuconazole, tricyclazole, and penconazole were used as azole fungicides in the selected areas according to a rotational method, in order to avoid the emergence of specific resistance to any active ingredient. A total of 84 soil and leaf samples were examined: 20 samples from vegetable and cereal fields, 24 samples from citrus groves and vegetable fields, 23 samples from vegetable fields, 10 samples from cereal fields, and seven samples from olive groves and cereal fields. All samples were collected in the period between March and October 2015.

### 2.2. Sample Treatment and Isolate Identification

All samples were treated according to the method described by Snelders et al. [17] with minor modifications. Briefly, 2 g of soil and pulverized leaves of each sample were suspended in 8 mL of 0.2 M NaCl solution with 1% Tween 20 (Sigma, St. Louis, USA) and vortexed. The suspension was maintained at room temperature for 30 minutes and 100 μL of supernatant was then inoculated on two plates of home-made Sabouraud dextrose agar (SDA, Biolife, Milan, Italy) supplemented with chloramphenicol (0.5 g/L, Sigma, Italy) and itraconazole (4 mg/L, Sigma, St. Louis, USA). Plates were then incubated at 32 °C and examined after 24, 48, and 72 hours of incubation. 

All isolates were identified by standard phenotypic methods, based on the macroscopic and microscopic morphological study of Czapek agar medium colonies (Difco, Becton Dickinson, Buccinasco, Italy). Only the itraconazole-resistant *Aspergillus* section *Fumigati* isolates were identified by sequencing. In particular, for the identification of *Aspergillus fumigatus sensu stricto,* a portion of the β-tubulin gene was amplified using the primer set Tub5 (5’-TGACCCAGCAGATGTT-3’) and Tub6 (5’-GTTGTTGGGAATCCACTC-3’) and analyzed by Sanger sequencing as described elsewhere [21]. The identification was confirmed if a 99–100% sequence identity was detected when the obtained sequences were compared, by using Basic Local Alignment Search tool (BLAST), to the sequences present in the GenBank database (www.ncbi.nlm.nih.gov).

### 2.3. Susceptibility Testing

The isolates grown on SDA supplemented with itraconazole were tested for antifungal susceptibility to itraconazole, voriconazole, and posaconazole by broth microdilution according to the European Committee on Antimicrobial Susceptibility Testing (EUCAST) method E:DEF 9.1 [22]. Minimum inhibitory concentration (MIC) values >2mg/L were used for defining resistance to itraconazole and voriconazole, while values >0.25 mg/L were used for defining resistance to posaconazole, according to EUCAST breakpoints [23,24,25]. In the absence of clinical breakpoints, the isolates of *Aspergillus* spp. were considered resistant when the MIC values were higher than the epidemiological cutoff value [26]. All assays were performed in duplicate, and *Aspergillus fumigatus* ATCC 204305 was included in each test as a quality-control strain. 

### 2.4. Detection of Mutation in Cyp51A Gene

All of the itraconazole-resistant *Aspergillus* section *Fumigati* isolates were subjected to *cyp51A* gene sequencing. Genomic DNA was extracted by using Dneasy plant tissue kit (Qiagen, Crawley, UK). The *cyp51A* coding region was amplified by PCR using the primer set P450-A1 (5’-ATGGTGCCGATGCTATGG-3) and Cyp51AR2 (5’-AGTGA ATAGAGGAGTGA ATCC-3’) and sequenced as described in Prigitano et al. [19]. The *cyp51A* gene promoter was amplified and sequenced using the primers P-A7 and P-A5 as previously described in Mellado et al. [27]. Sequence alignment was performed using the ClustalW algorithm (www.ebi.aci.uk). The *cyp51A* sequence from *A. fumigatus* strain 237 (GenBank accession number: AF338659) was used as a wild type reference.

## 3. Results

*Aspergillus* spp. grew on SDA supplemented with itraconazole in 20 of 84 leaf and soil samples (20/84, 23.8%). *Aspergillus* section *Fumigati* grew in five of 84 samples (5/84, 5.9%) and was not isolated in samples from the olive groves and cereal fields of Basilicata and from the citrus groves and vegetable fields of Sicily. No *Aspergillus* spp. were isolated from the sampled olive groves and cereal fields of Basilicata (Table 1).

Susceptibility testing performed by broth microdilution on the five *Aspergillus* section *Fumigati* isolates confirmed itraconazole resistance in four isolates (4/84, 4.7%) with a range of MIC values from 8 to >16 mg/L, while no resistance was confirmed for the fifth isolate (MIC=0.125 mg/L). These four isolates (S-15, C-63, C-68, and S-80) were all identified as *A. fumigatus sensu stricto* by amplification and sequencing of a fragment of the β-tubulin gene. Three of them (S-15, C-68, and S-80), (3/84, 3.6%), were posaconazole resistant (range of MICs: 0.5–2 mg/L) and two (C-68 and S-80), (2/84), 2.4%) were voriconazole resistant (range of MICs: 4–8 mg/L). Sequence analysis of the *cyp51A* gene showed the t364a point mutation, which results in the L98H substitution, combined with the 34-base pair tandem repeat in the promoter region, in S-15, C-68, and S-80 isolates, while no TR_34_/L98H mutation in the *cyp51A* gene was found in the C-63 isolate (Table 2). 

The *Aspergillus* spp. isolates grown on SDA containing itraconazole were morphologically identified as follows: nine isolates of *Aspergillus* section *Nigri*, three isolates of *Aspergillus* section *Flavi*, and one isolate of *Aspergillus* section *Terrei.* In 11 of the 15 *Aspergillus* spp. isolates, itraconazole resistance was confirmed by broth microdilution: according to the EUCAST method, all *Aspergillus* section *Nigri* isolates (9/84, 10.7%) were considered itraconazole-resistant, as they displayed MIC values (range of MICs: 8 to >16 mg/L) higher than the epidemiological cutoff value (4 mg/L); two *Aspergillus* section *Flavi* isolates (2/84, 2.4%) were itraconazole-resistant (range of MICs: 4–8 mg/L), and one showed reduced susceptibility to itraconazole (MIC value = 2 mg/L); and the *Aspergillus* section *Terrei* isolate showed reduced susceptibility to itraconazole (MIC value = 2 mg/L). Only four *Aspergillus* section *Nigri* isolates exhibited MIC values for voriconazole higher than the epidemiological cutoff value (4 mg/L) and could be considered as voriconazole-resistant. MIC values for posaconazole were as follows: 0.125–1 mg/L for *Aspergillus* section *Nigri* isolates; 0.06–0.125 mg/L for *Aspergillus* section *Flavi* isolates; and 0.125 mg/L for the *Aspergillus* section *Terrei* isolate (Table 3). 

## 4. Discussion

The azole resistance of *Aspergillus* spp. is an increasing global health concern, and is most frequently observed in European countries [16,17,28,29]. Two routes of resistance development are generally described: long-term azole patient therapy in clinical settings, and following the application of azole compounds in agriculture. The environmental usage of triazole in agriculture has been suggested as playing an essential role in the development and spread of resistance to medical triazole in *Aspergillus* species [15,30]. Azole cross-resistance is also a growing concern. Several studies, for example, have demonstrated that isavuconazole highly correlates with voriconazole for *A. fumigatus,* while the eventual cross-resistance pattern is not well known for other *Aspergillus* species [31,32].

In the present study, we evaluated the prevalence of resistance to triazoles in *Aspergillus* spp. and *Aspergillus* section *Fumigati* strains isolated from agricultural areas in Southern Italy. We identified four azole-resistant *A. fumigatus sensu stricto* isolates, and three of them carried the TR_34_/L98H resistance mechanism. Although TR_34_/L98H represents the predominant mutation in azole-resistant *A. fumigatus* strains, several resistance mechanisms have been described [28,31], so the recovery of an isolate with an azole-resistant phenotype but no mutations in the *cyp51A* gene is not surprising. The isolation of *A. fumigatus* strains harboring the sole TR_34_/L98H resistance mechanism could also be attributable to the use, for the initial screening, of only itraconazole-containing agar. This could result in the eventual exclusion of voriconazole-resistant strains that are not necessarily itraconazole resistant, since other azole resistance mechanisms could be involved (e.g., TR_46_/Y121F/T289A) [30,33]. In future studies, the use of itraconazole- and voriconazole-containing plates could probably help to widen the range of azole-resistant isolates to obtain further and more detailed information. In our findings, in 4.7% (4/84) of the samples, azole-resistant *A. fumigatus sensu stricto* isolates were found, 4.7% (4/84) were itraconazole-resistant, 3.6% (3/84) were posaconazole-resistant, and 2.4% (2/84) were voriconazole resistant. Looking at the occurrence of azole-resistant *A. fumigatus* isolates across Europe, in the present study we found lower frequencies of resistant isolates compared to Italy (16.9% for *A. fumigatus sensu stricto*) [20] and other European countries such as Denmark (8% for *A. fumigatus*) [18] and the Netherlands (20.4% for *A. fumigatus*) [17]. These observations could probably be related to the isolation, in the environment of the sampled areas of Southern Italy, of a large number of non-*fumigatus Aspergillus* spp. (*Aspergillus* section *Nigri*, *Aspergillus* section *Flavi,* and *Aspergillus* section *Terrei*), which were itraconazole resistant: we found in particular 13.1% (11/84) itraconazole-resistant isolates, 2.4% (2/84) isolates with reduced susceptibility to itraconazole, and 4.76% (4/84) voriconazole-resistant isolates. Moreover, these observations could also depend on the fact that the study was designed to screen for resistant *Aspergillus* and consequently lacked the total number of *Aspergillus* isolates recovered (resistant and sensitive isolates). In conclusion, this study provides evidence for a high prevalence of phenotypic azole resistance in non-*fumigatus Aspergillus* spp. isolated from the environment in several areas of Southern Italy. These observations might be very useful to study the possible mechanisms underlying azole resistance in these other *Aspergillus* species which, although less pathogenic than *A. fumigatus*, are very frequently isolated in many areas of Southern Italy and in particular in Sicily, representing a potential cause of invasive disease in patients exposed to conidia from the environment.

In future studies, a surveillance program is needed to monitor the emergence of environmental multi-azole resistance, which could have important implications for the management of patients with *Aspergillus* diseases. Since early diagnosis and effective treatment of azole-resistant aspergillosis still represent a challenge, monitoring both clinical and environmental isolates would significantly contribute to a better understanding of the epidemiology and origin of azole-resistant *Aspergillus* spp. in the context of an inevitable battle against this global health problem.

## Figures and Tables

**Table 1:** Environmental origin of itraconazole-resistant *Aspergillus* spp. and *Aspergillus* section *Fumigati* isolates.

Sample Source	Region	Examined Samples (*n*)	Samples with Growth of *Aspergillus* spp.* on SDA Containing Itraconazole (*n*)	Samples with Growth of *Aspergillus* Section *Fumigati* on SDA Containing Itraconazole (*n*)	*Aspergillus* spp.* Isolates with Confirmed Itraconazole Resistance (*n*) **	*A. fumigatus sensu stricto* Isolates with Confirmed Itraconazole Resistance (*n*)
Vegetable fields	Sicily (S)	23	6	2	5	1
Citrus groves and cereal fields	Sicily (S)	24	5	0	4	0
Olive groves and cereal fields	Basilicata (B)	7	0	0	0	0
Vegetable and cereal fields	Calabria (C)	20	3	2	1	2
Cereal fields	Sicily (S)	10	1	1	3	1
Total	-	84	15	5	11	4

* *Aspergillus* section *Terrei, Aspergillus* section *Flavi,* and *Aspergillus* section Nigri. ** In the absence of clinical breakpoint, the isolates of *Aspergillus* spp. were considered itraconazole resistant when the minimum inhibitory concentration (MIC) values were higher than the epidemiological cutoff value.

**Table 2:** Results of susceptibility testing performed by broth microdilution (EUCAST) and analysis of *cyp51A* mutations for four azole-resistant *A. fumigatus sensu stricto* isolates.

Isolate Number	Fungicides Used	Sample Source	MIC (mg/L) Determined by EUCAST			Detection of *Cyp51A* Mutations
			Itraconazole	Posaconazole	Voriconazole	
S-15	Bromuconazole Tetraconazole Penconazole	Vegetable field	>16	2	1	TR_34_/L98H
C-63	Bromuconazole Difenoconazole Hexaconazole	Vegetable and cereal fields	8	0.25	0.5	None
C-68	Bromuconazole Difenoconazole Hexaconazole	Vegetable and cereal fields	>16	0.5	4	TR_34_/L98H
S-80	Bromuconazole Cyproconazole Fenbuconazole	Cereal field	>16	2	8	TR_34_/L98H

Itraconazole clinical breakpoint S ≤ 1 and R > 2 mg/L; posaconazole clinical breakpoint S ≤ 0.125 and R > 0.25 mg/L; voriconazole clinical breakpoint S ≤ 1 and R > 2 mg/L.

**Table 3:** Results of EUCAST (European Committee on Antimicrobial Susceptibility Testing) susceptibility testing for 13 *Aspergillus* spp. isolates.

Identified Isolate	Isolate Number	Sample Source	MIC (mg/L) Determined by EUCAST		
			Itraconazole	Posaconazole	Voriconazole
*Aspergillus* section *Nigri*	S-2	Vegetable fields	8	0.25	2
	S-7	Vegetable fields	8	0.25	1
	S-11	Vegetable fields	>16	0.5	4
	S-19	Vegetable fields	>16	0.5	4
	S-25	Citrus groves and vegetable fields	8	0.125	1
	S-31	Citrus groves and vegetable fields	8	0.125	2
	S-40	Citrus groves and vegetable fields	8	0.125	1
	S-75	Cereal fields	>16	0.25	4
	S-77	Cereal fields	>16	1	4
*Aspergillus* section *Flavi*	S-3	Vegetable fields	4	0.06	0.5
	S-29	Citrus groves and vegetable fields	2	0.06	0.25
	C-58	Vegetable and cereal fields	8	0.125	0.5
*Aspergillus* section *Terrei*	S-81	Cereal fields	2	0.125	0.5

Itraconazole/*A. flavus* clinical breakpoint S ≤ 1 and R > 2 mg/L; itraconazole/*A. terreus* clinical breakpoint S ≤ 1 and R > 2 mg/L; itraconazole/*A. niger* epidemiological cutoff value (ECOFF): 4 mg/L; posaconazole/*A. terreus* clinical breakpoint S ≤ 0.125 and R > 0.25 mg/L; posaconazole/*A. flavus* and *A. niger* no clinical breakpoint or ECOFF; voriconazole/*A. niger* ECOFF: 2 mg/L; voriconazole/*A. flavus* ECOFF: 2 mg/L; voriconazole/*A. terreus* ECOFF: 2 mg/L.
